# A case report of giant inflammatory mass in the hypopharynx

**DOI:** 10.1097/MD.0000000000050548

**Published:** 2026-09-04

**Authors:** Fengjin Chu, Demin Li, Yuhui Ren, Xu Ma, Jin Zhang, Yungang Wu

**Affiliations:** a Clinical Medical College of Jining Medical University, Jining, Shandong, China; b Department of Otolaryngology-Head and Neck Surgery, Affiliated Hospital of Jining Medical University, Jining, Shandong, China.

**Keywords:** enteral nutrition, hypopharynx, inflammatory mass, intratracheal intubation

## Abstract

**Rationale:**

Hypopharyngeal inflammatory masses are rare conditions that may occur in association with factors such as intubation, trauma, or reflux. Their clinical and imaging features often closely mimic those of malignant tumors, necessitating a definitive diagnosis through the histopathological examination of surgical specimens. Although surgical excision remains the primary intervention for large lesions, vigilant postoperative surveillance is imperative because of the high recurrence rates.

**Patient concerns:**

We detail the case of a 26-year-old female admitted for a traumatic brainstem injury. Following 21 days of endotracheal intubation and over 2 months of nasogastric tube placement, flexible laryngoscopy revealed a mass in the left pyriform sinus, prompting referral to the Department of Otolaryngology-Head and Neck Surgery. The patient exhibited dysphagia and mild hoarseness as her sole symptoms.

**Diagnoses:**

Computed tomography and neck magnetic resonance imaging demonstrated a mass measuring approximately 32 mm × 27 mm at its largest cross-section, with ill-defined borders, heterogeneous density, and multiple bilateral cervical lymph nodes. Biopsy revealed inflammatory necrosis and granulation tissue.

**Interventions:**

Given the large size of the mass, persistent dysphagia, the inability to completely exclude malignancy based on imaging findings, and limited biopsy results, complete resection was performed under general anesthesia to alleviate symptoms and establish a definitive diagnosis. Subsequent pathological examination confirmed the presence of an inflammatory mass composed of necrotic and proliferative granulation tissue.

**Outcomes:**

The patient recovered well after the surgical removal of the lesion. During the 4-month follow-up period, a laryngoscopy performed 11 weeks after surgery revealed no recurrence of the mass. At the final telephone follow-up at 4 months after surgery, the patient reported no throat-related symptoms.

**Lessons:**

This case suggests that prolonged tube placement may be a potential contributing factor in the development of inflammatory masses in the pyriform sinus. In patients with a history of long-term tube placement who present with a pyriform sinus mass, inflammatory masses may be considered among the differential diagnoses.

## 1. Introduction

Benign space-occupying lesions of the hypopharynx are relatively rare, and inflammatory masses are particularly uncommon. The pyriform sinus is the most common site of hypopharyngeal carcinoma (accounting for approximately 65%–85% of cases).^[1]^ Malignancy is often the first consideration for space-occupying lesions in this region. However, a small number of benign inflammatory lesions may also present as mass-like proliferations, making it difficult to distinguish them from hypopharyngeal carcinomas, both clinically and on imaging. This report describes a case of an inflammatory mass originating in the pyriform sinus and, in conjunction with a review of the literature, discusses its clinical characteristics and diagnostic and therapeutic strategies to enhance awareness of this rare benign lesion and facilitate accurate diagnosis.

## 2. Case report

### 2.1. Patient information

A 26-year-old female was transferred to our intensive care unit on January 27, 2025, after resuscitation and subsequent intubation at a peripheral hospital for the management of a traumatic craniocerebral injury with brainstem involvement. On admission, she was comatose, with sluggish pupillary reflexes, bilateral Babinski signs, and poor responsiveness to stimuli. A standardized neurocritical care bundle encompassing nasogastric tube placement, mechanical ventilation, measures to lower intracranial pressure, and neuroprotective therapy was immediately established.

On post-intubation day 21 (February 17, 2025), the patient exhibited eye opening in response to noxious stimuli while maintaining spontaneous respiration, which prompted tracheostomy. Throughout the subsequent neurorehabilitation, her conscious state evolved to full alertness, with the capacity to follow simple commands, culminating in brief assisted wheelchair sitting. By March 25, she had developed new-onset dysphagia and dysarthria, necessitating targeted swallowing and speech rehabilitation. On April 7, after more than 2 months of nasogastric tube feeding, she successfully resumed oral intake, allowing removal of the nasogastric tube. Her functional status and limb muscle strength continued to improve with ongoing rehabilitation, and she maintained a normal diet.

### 2.2. Diagnostic assessment

**Figure 1.**
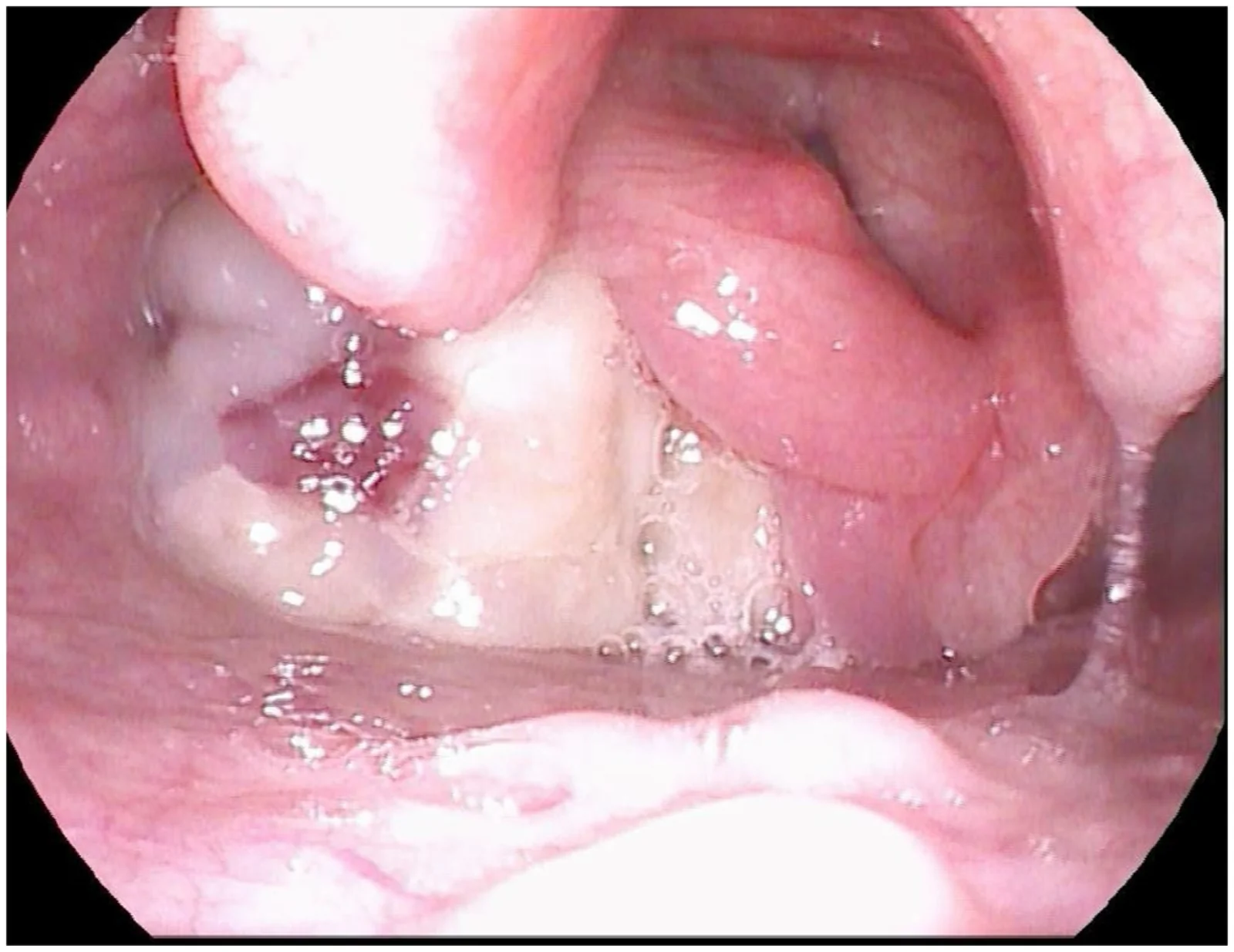
Laryngoscopic findings. A mass in the left pyriform sinus. The lesion was yolk-sized, irregular, firm, and presented with a rough, grayish-white surface covered with adherent white secretions.

A tube occlusion test was initiated on April 9 to gradually close the tracheostomy cannula opening and assess the patient’s ability to breathe spontaneously through the mouth and nose. During the 72-hour continuous tube occlusion period, the patient showed no signs of dyspnea or respiratory distress during or after activity. On April 12, a flexible laryngoscopy revealed a new growth in the left pyriform sinus. The mass was egg-sized and irregular, with a firm texture. Its surface appeared rough and grayish-white and was associated with adherent white secretions. Bilateral vocal cord mobility was reduced (Fig. 1). However, there was no significant narrowing of the central or glottic airways, which met the criteria for decannulation, and the tracheostomy tube was successfully removed.

**Figure 2.**
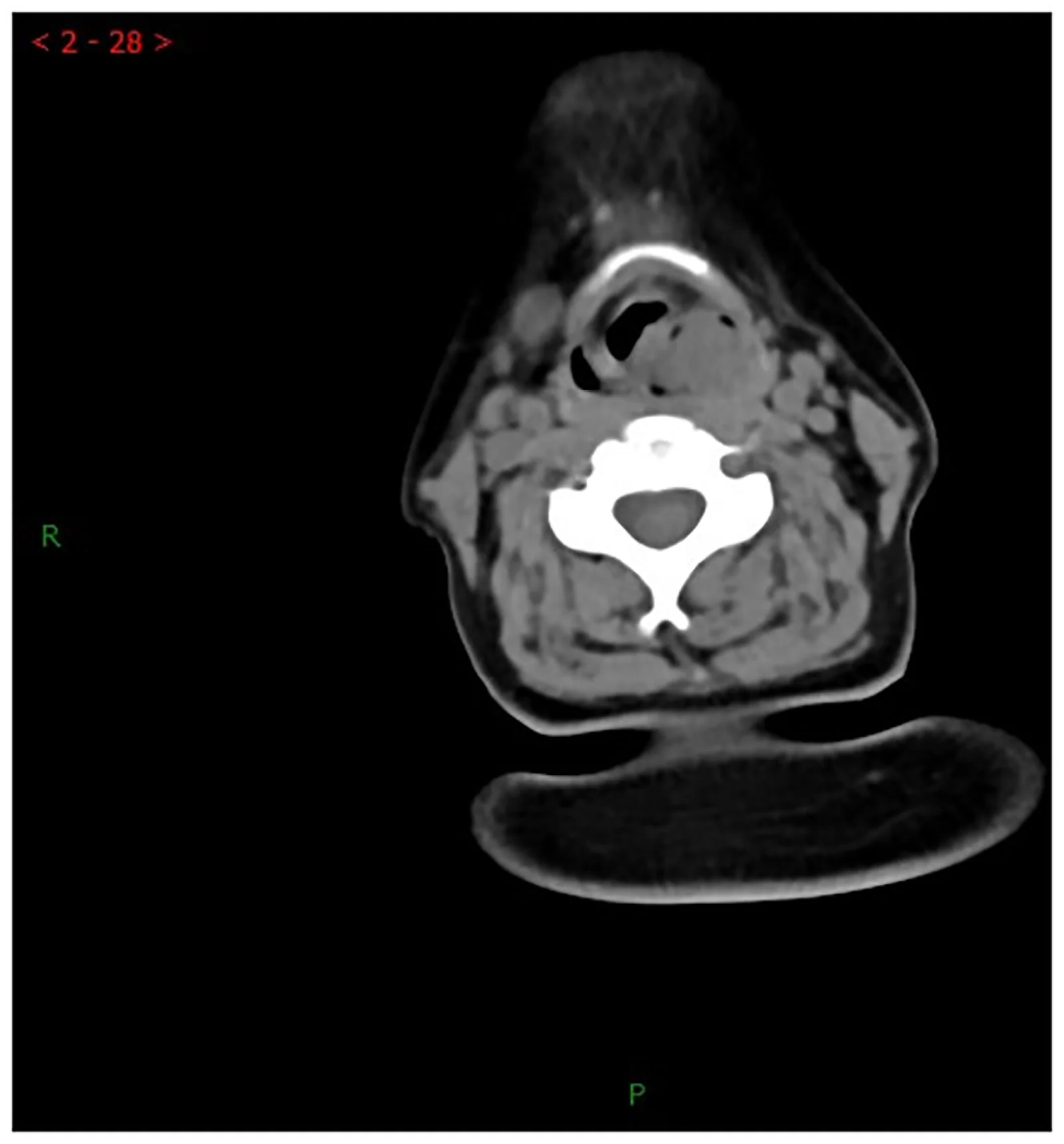
Neck CT findings. An ill-defined, heterogeneously hypodense mass in the left pyriform sinus, measuring 32 mm × 27 mm. CT = computed tomography.

**Figure 3.**
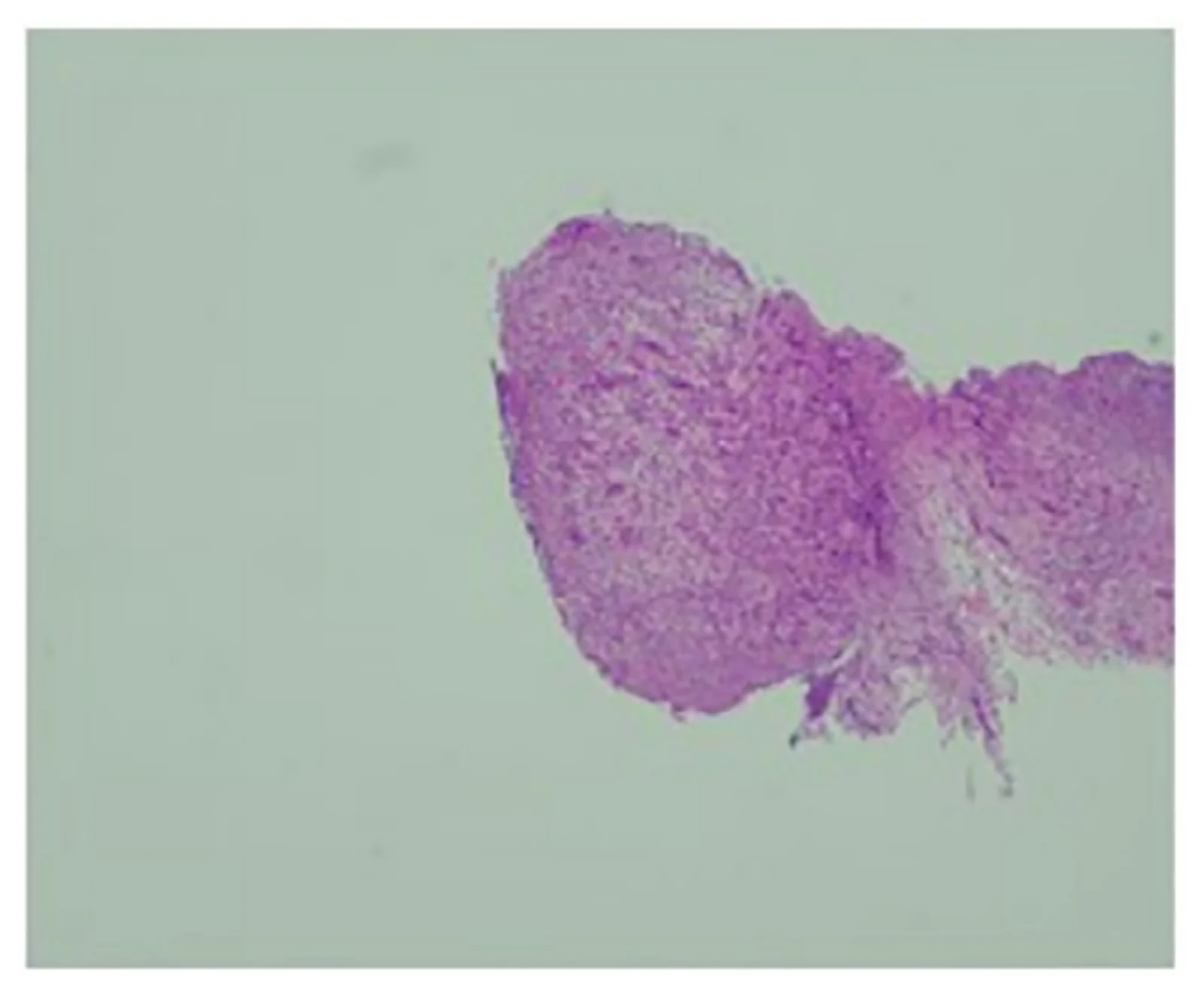
Histopathological image of the biopsy. Inflammatory granulation tissue and necrosis within the hypopharyngeal mass.

Computed tomography (CT) revealed a heterogeneously hypodense soft-tissue mass in the left pyriform sinus, measuring 32 mm × 27 mm in its greatest axial dimension with ill-defined margins. Multiple bilateral cervical lymph nodes with homogeneous enhancement were observed. Given the irregular shape and blurred borders of the lesion, CT findings suggested a neoplastic lesion (Fig. 2). A 1-week therapeutic trial with continued omeprazole (for reflux) and escin (for edema reduction) resulted in no decrease in the size of the mass, prompting a biopsy. Multiple samples were obtained using biopsy forceps under direct laryngoscopic visualization; the specimens were rice-grain-sized and contained an adequate amount of tissue, inflammatory granulation tissue, and necrosis within the hypopharyngeal mass (Fig. 3).

**Figure 4.**
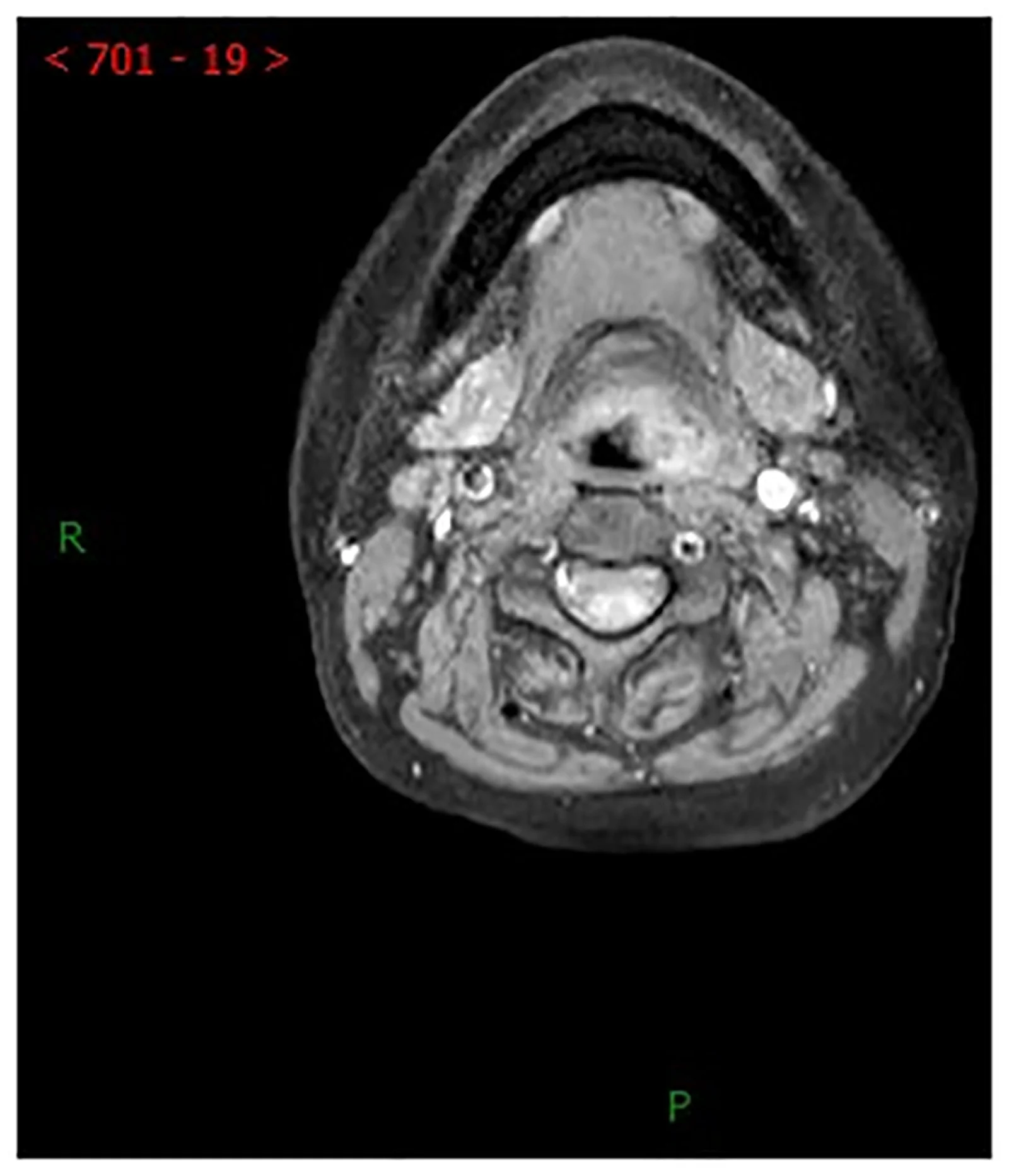
Neck MRI findings. A mass in the left pyriform sinus is shown. MRI = magnetic resonance imaging.

Due to sampling errors inherent in single-site biopsies, it was difficult to completely rule out occult lesions deep within the mass. Given the high suspicion of malignancy on CT and persistent dysphagia, surgical resection is recommended. Consequently, on May 6, the patient was transferred to the Department of Otolaryngology and Head and Neck Surgery. Neck magnetic resonance imaging performed on May 7 revealed an irregular nodule within the left pyriform sinus. The lesion was hypointense on T1-weighted imaging, hyperintense on T2-weighted imaging and diffusion-weighted imaging, and showed heterogeneous postcontrast enhancement. Multiple bilateral cervical lymph nodes showed homogeneous enhancement. The typical radiological findings described above (limited diffusion, heterogeneous enhancement, and regional lymph node enlargement) raised suspicion of malignancy with possible inflammatory changes (Fig. 4).

### 2.3. Therapeutic intervention

**Figure 5.**
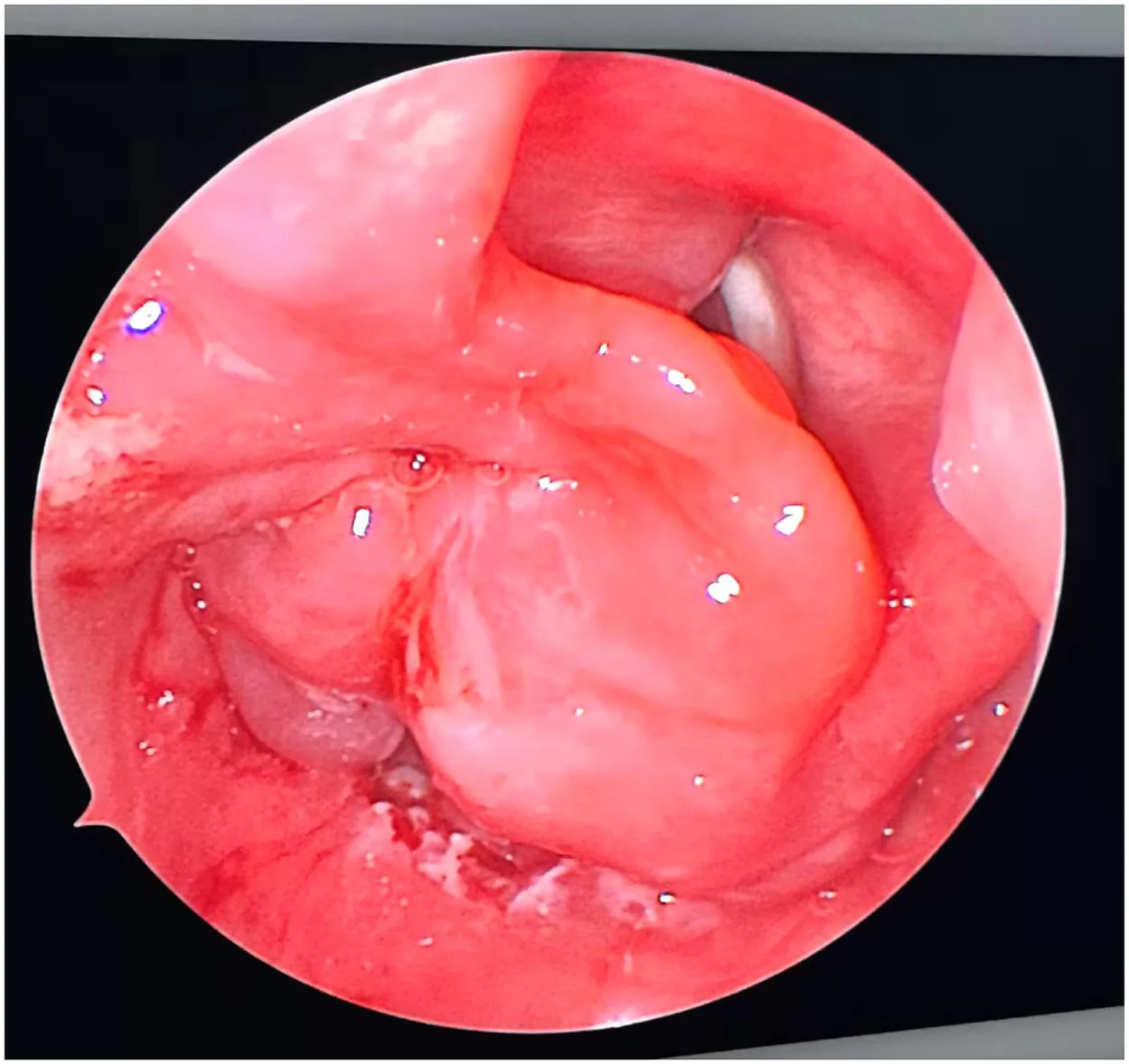
Transoral resection of a left pyriform sinus mass under suspension laryngoscopy.

**Figure 6.**
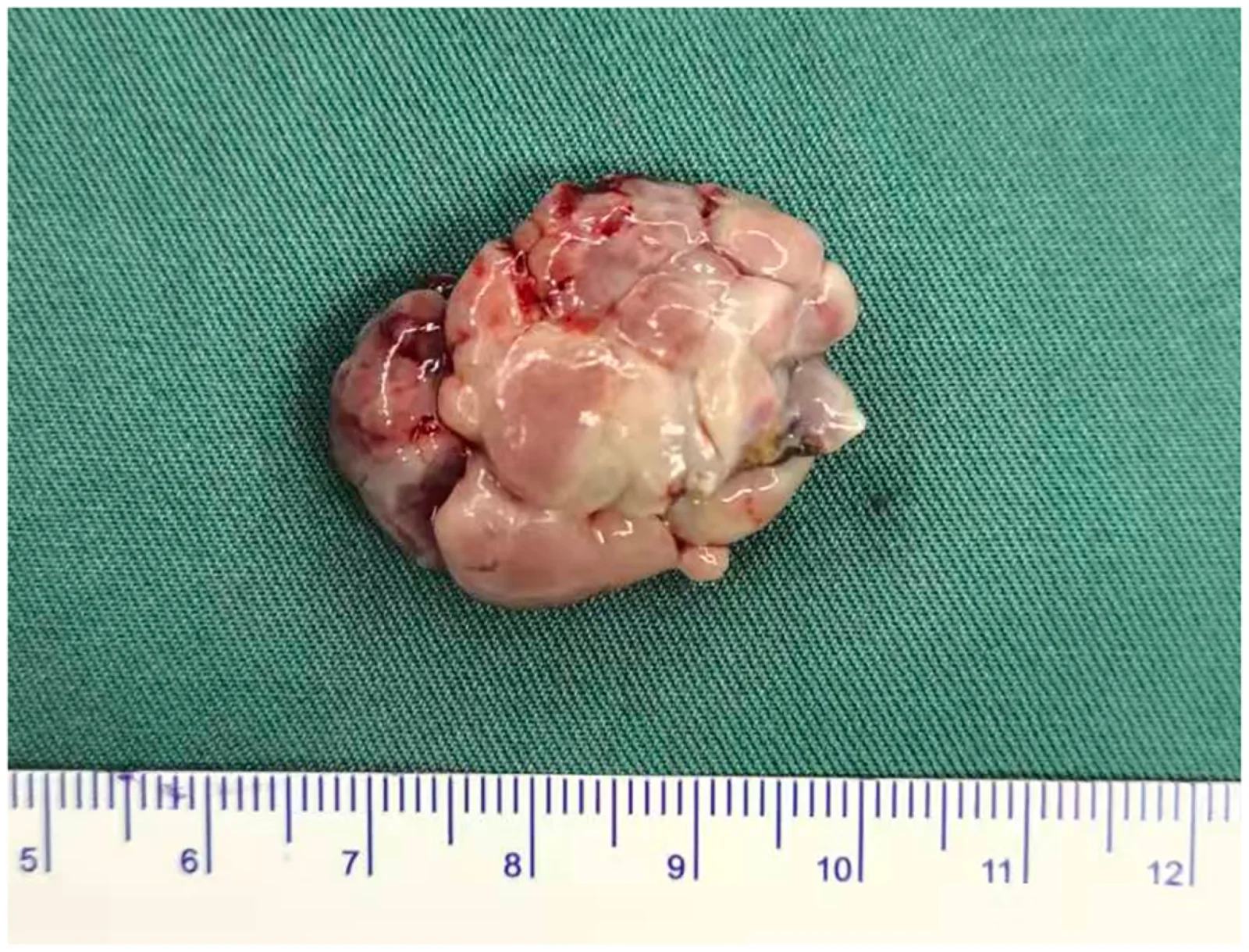
Gross photograph of the resected specimen. The mass is lobulated and measures 3.5 cm in maximal diameter. It has a firm consistency, a grayish-white appearance, and a pedicle arising from its base.

**Figure 7.**
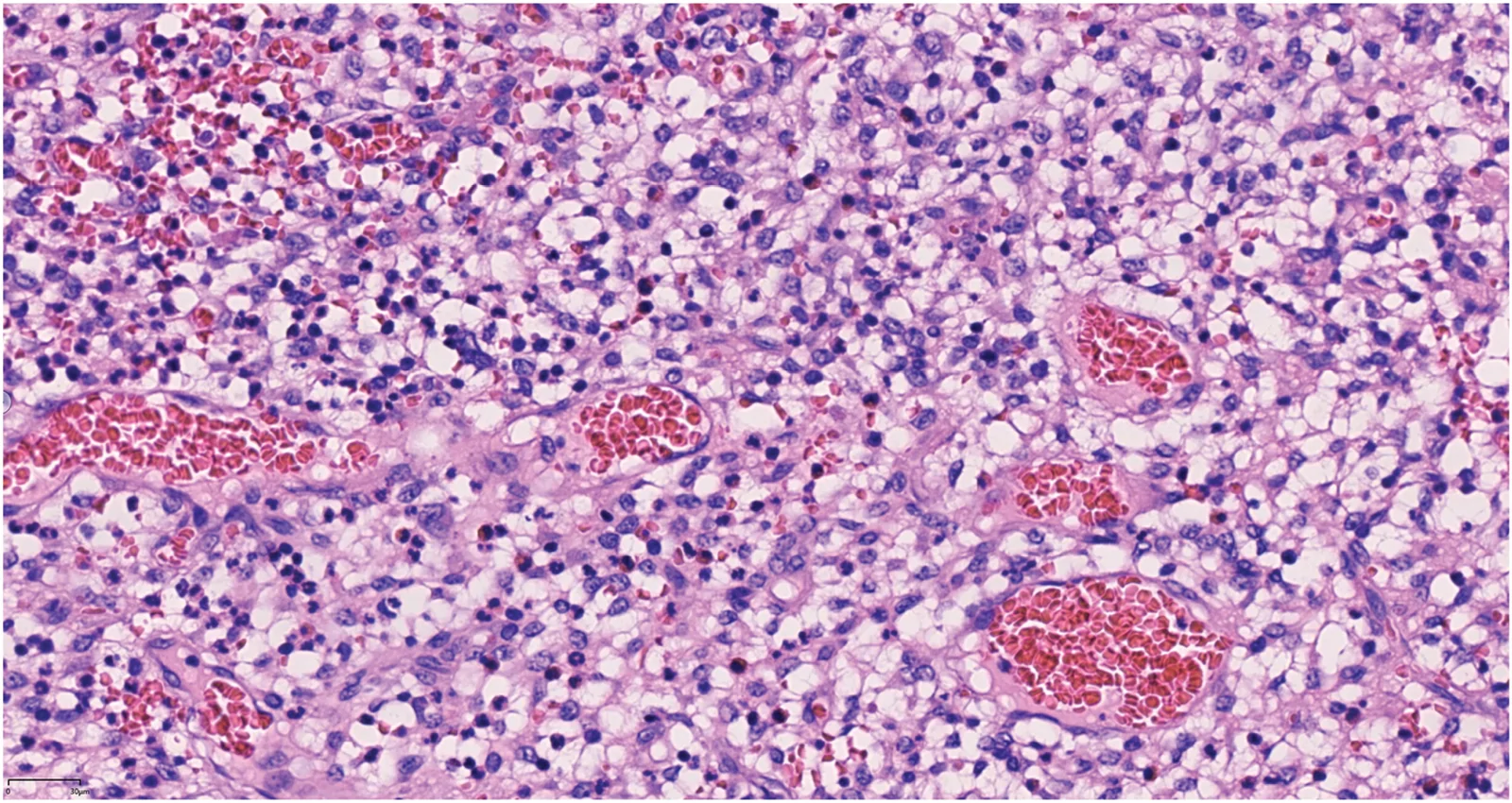
Histopathological findings of the hypopharyngeal mass. HE staining, ×40 magnification, showing inflammatory granulation tissue and necrosis. HE = hematoxylin and eosin.

Given the large size of the lesion, persistent dysphagia, inability to completely exclude malignancy based on imaging findings, and limited biopsy results, surgical excision was performed to obtain a definitive diagnosis and relieve the symptoms. Intraoperatively, a large lobulated mass was visualized in the left pyriform sinus, measuring approximately 3.5 cm in maximal diameter (yolk size), with firm consistency and a grayish-white appearance. Its base extended to the arytenoid muscle with a pedicle arising from the medial wall of the left pyriform sinus. The lesion was completely excised intraoperatively using a low-temperature plasma knife, with a 3-mm margin maintained around the lesion. A 3-mm margin was selected because the diameter of the plasma knife tip was 3 mm. The resected specimen was further submitted for intraoperative frozen section examination, which revealed necrotic tissue and proliferative granulation tissue without malignant components. Therefore, the resection range was not further extended. The postoperative wound measured 0.5 cm × 0.5 cm and did not require sutures (Figs. 5 and 6). Postoperative paraffin-embedded histopathology confirmed extensive necrotic and proliferative granulation tissue with inflammatory cell infiltration, without malignant cells (Fig. 7). Special staining and immunohistochemical analyses were not performed because routine histopathological findings were sufficient to establish a diagnosis. No otolaryngological-specific postoperative management was required. The patient was prescribed a 3-month course of antireflux therapy with omeprazole and was transferred back to the Department of Rehabilitation for ongoing traumatic brain injury management.

### 2.4. Outcomes

**Figure 8.**
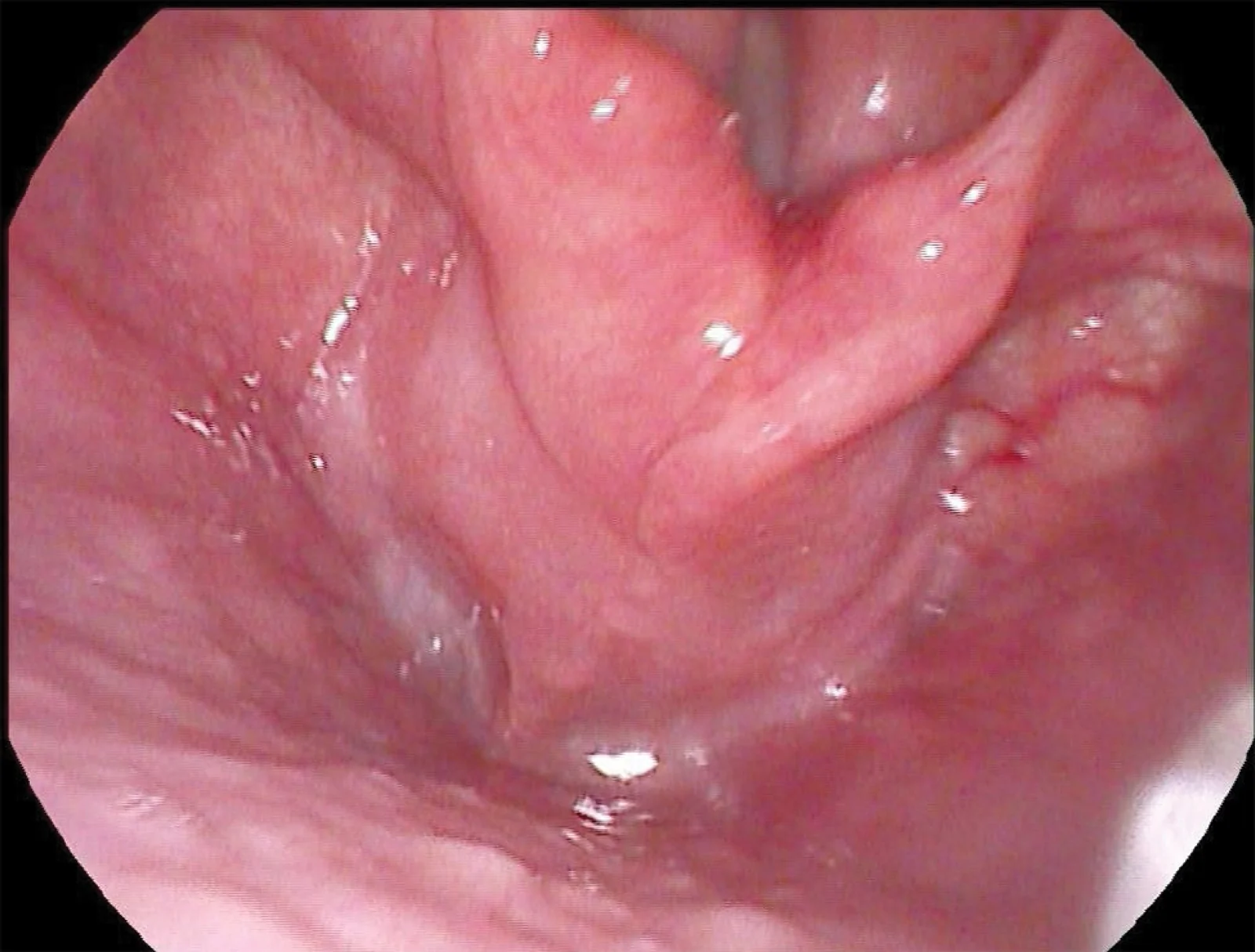
Follow-up laryngoscopy at 11 weeks postoperatively (July 24, 2025). The image demonstrates complete mucosal healing at the resection site in the left piriform sinus, with no evidence of residual lesion or granulation tissue.

During the 4-month follow-up period, surveillance laryngoscopy performed on July 24, 2025, demonstrated mucosal healing in the left pyriform sinus (Fig. 8). Postoperatively, the patient’s swallowing function, hoarseness, airway discomfort, and ability to eat by mouth improved compared to preoperative levels, and the mobility of both vocal cords partially recovered. At the 4-month postoperative telephone follow-up, the patient reported no significant discomfort.

The timeline is summarized in Table 1.

**Table 1:** Timeline of clinical events.

Date	Clinical event
January 27, 2025	Endotracheal intubation, ICU admission, and nasogastric tube placement after traumatic brainstem injury
February 17, 2025	Tracheostomy performed
March 25, 2025	Dysphagia and dysarthria developed
April 7, 2025	Nasogastric tube removed
April 12, 2025	Laryngoscopy revealed a mass in the left pyriform sinus; tracheostomy decannulation performed
April 22, 2025	Laryngoscopic biopsy performed; pathology revealed inflammatory granulation tissue and necrosis
May 9, 2025	Complete surgical excision of the mass performed
July 24, 2025	Follow-up laryngoscopy (11 weeks after surgery) showed no recurrence
4 months after surgery	Telephone follow-up revealed no throat-related symptoms

ICU = intensive care unit.

## 3. Discussion

The inflammatory response is a nonspecific defense reaction of the body to exogenous (infectious, physical, or traumatic) or endogenous (immune-related) insults. In most cases, the inflammatory response is a protective reaction that helps eliminate pathogens and stimulates repair of damaged tissues.^[2]^ However, when the inflammatory response persists or becomes excessive, proliferative changes may occur in local tissues, and in some cases, macroscopically visible inflammatory masses may form. Notably, inflammatory masses localized in the hypopharynx, particularly in the pyriform sinus, are exceedingly rare.

The uniqueness of this case stems from its clinical and radiological presentation, which closely mimicked that of a malignancy. The lesion observed in this case was located in the pyriform sinus and measured 32 mm × 27 mm. Its irregular shape and rough surface made it extremely difficult to distinguish it from pyriform sinus carcinoma. Clinically, early hypopharyngeal cancer often presents as globus pharyngeus. As the tumor enlarges, patients may develop dysphagia or a choking sensation, all of which are consistent with the symptoms observed in our case. Radiologically, imaging findings were highly suggestive of malignancy, making it impossible to rule it out.^[3]^ In addition, the patient received omeprazole for acid suppression, commencing with nasogastric tube insertion. Following mass detection, sodium aescinate was supplemented for 1 week to reduce swelling, yet no mass reduction occurred. Given the potential for biopsy sampling errors and the possibility of malignant tumors developing into secondary inflammatory mass formation, surgical resection for a definitive histopathological diagnosis is imperative.

The inflammatory mass in the pyriform sinus described in this case should be differentiated from several other inflammatory and benign hypopharyngeal lesions. Post-intubation granuloma is an important differential diagnosis. It is a benign inflammatory proliferative lesion caused by chronic mucosal trauma after endotracheal intubation and usually occurs in the posterior laryngeal region, such as the vocal process or cuneiform area. Although the lesion in our case was located in the pyriform sinus rather than in the typical laryngeal region, the patient’s history of prolonged endotracheal intubation and nasogastric tube placement suggested a possible mechanism similar to that of persistent mucosal irritation. Previous studies have described the association of hypopharyngeal granulomas with prolonged airway instrumentation.^[4,5]^ Inflammatory pseudotumor should also be considered. It is a tumorlike inflammatory lesion characterized by myofibroblastic proliferation and inflammatory cell infiltration, which may show local invasiveness. In contrast, histopathological examination of our patient revealed necrosis with proliferative granulation tissue without spindle cell proliferation, suggesting an inflammatory pseudotumor.^[6]^ Infectious granulomatous diseases and foreign-body reactions represent additional differential considerations. These lesions are usually associated with specific pathogens or retained foreign materials, and may demonstrate granulomatous inflammation or foreign-body giant cell reactions upon pathological examination. No evidence of infection or foreign-body reactions was observed in the specimens.^[7]^ Other benign hypopharyngeal masses including inflammatory polyps, papillomas, and fibrolipomas may present as mass-like lesions. However, their clinical and pathological characteristics differed from those observed in the present case.

The pathogenesis of inflammatory masses is likely multifactorial. In the present case, prolonged intubation, nasogastric tube placement, and reflux may have acted as potential contributors to local mucosal irritation. Santos et al reported that the presence of nasogastric and endotracheal tubes, the duration of intubation, and tube dimensions were associated with the development of laryngeal granulomas.^[8–10]^ Sarah et al reported an esophageal inlet granuloma in a patient with a history of 6 weeks of nasogastric tube placement. In our case, both nasogastric and endotracheal tubes may have contributed to mucosal irritation in the hypopharynx and pyriform sinus.^[11]^ Concurrently, nasogastric tube placement may impair esophageal sphincter function, potentially leading to reflux of gastric and esophageal contents. Such inflammatory irritation and reflux may exacerbate local inflammatory responses, potentially contributing to inflammatory mass formation.

Regarding the management of inflammatory proliferative lesions, Rimoli et al reported that antibiotics, zinc sulfate, acid suppression therapy, and surgical intervention have been employed to prevent or treat post-extubation laryngeal granuloma formation. Although these reports focused mainly on laryngeal granulomas, they provide references for the management of inflammatory proliferative lesions. However, the efficacy of these treatments varies, and no first-line treatment has yet been established.^[4]^ Santos et al indicate that smaller laryngeal granulomas may resolve spontaneously within 8 to 14 weeks post-extubation. Surgical excision may be considered for larger granulomas or those that are unresponsive to medical therapy.^[8,12]^ In this case, surgical intervention was considered appropriate because of the patient’s marked dysphagia, large mass, lack of response to acid-suppressing and anti-edema therapies, and the inability of imaging studies to exclude malignancy.

## 4. Limitations

This study has several limitations. First, it involved only a single case, the follow-up period was relatively short, and the final follow-up was conducted by telephone. Second, although potential contributing factors were discussed, a causal relationship between these factors and lesion development could not be established. Finally, additional pathological investigations, such as special staining and immunohistochemical analysis, were not performed.

## 5. Conclusion

A hypopharyngeal inflammatory mass is a rare inflammatory lesion that is infrequently documented in the literature because of its uncommon location. Potential contributing factors may include prolonged endotracheal intubation, nasogastric tube placement, and gastroesophageal reflux. Clinically and radiologically, this lesion can be mistaken for cancer; therefore, a definitive diagnosis requires histopathological examination of the excised tissue. Initial management typically involves conservative measures, such as acid suppression therapy. For lesions with pronounced symptoms, large size, and uncertain malignant potential, surgical intervention may be considered. Given the possibility of recurrence and limited follow-up duration in this case, long-term follow-up is advisable.

## Acknowledgments

All the authors thank Jining Medical University and its Affiliated Hospital for providing the learning platform and also express our gratitude to the corresponding authors for the careful guidance on the report.

## Author contributions

**Data curation:** Fengjin Chu, Yuhui Ren, Xu Ma.

**Investigation:** Fengjin Chu, Yuhui Ren, Xu Ma.

**Supervision:** Jin Zhang, Yungang Wu.

**Validation:** Jin Zhang, Yungang Wu.

**Funding acquisition:** Yungang Wu.

**Writing – original draft:** Fengjin Chu, Demin Li.

**Writing – review & editing:** Jin Zhang, Yungang Wu.
